# Vivax malaria in Mauritania includes infection of a Duffy-negative individual

**DOI:** 10.1186/1475-2875-10-336

**Published:** 2011-11-03

**Authors:** Nathalie Wurtz, Khadijetou Mint Lekweiry, Hervé Bogreau, Bruno Pradines, Christophe Rogier, Ali Ould Mohamed Salem Boukhary, Jamal Eddine Hafid, Mohamed Salem Ould Ahmedou Salem, Jean-François Trape, Leonardo K Basco, Sébastien Briolant

**Affiliations:** 1 Unité de Recherche en Biologie et Epidémiologie Parasitaires, Institut de Recherche Biomédicale des Armées, Allée du médecin colonel Eugène Jamot, Parc du Pharo, BP 60109, 13262 Marseille Cedex 07, France; 2 Unité de Recherche sur les Maladies Infectieuses et Tropicales Emergentes, UMR 6236, Marseille, France; 3 Laboratoire de Biotechnologies, Faculté des Sciences et Techniques, Université de Nouakchott, Mauritania; 4 UFR Biologie et Santé, Département de Biologie, Faculté des Sciences Semlalia, Université Cadi Ayyad, Marrakech, Morocco; 5 Laboratoire Aliments, Environnement et Santé (LAES), Faculté des Sciences et Techniques, Université Cadi Ayyad, Marrakech, Morocco; 6 Institut Pasteur de Madagascar, Antananarivo, Madagascar; 7 Laboratoire de Paludologie, Institut de Recherche pour le Développement, Dakar, Sénégal

**Keywords:** *Plasmodium vivax*, Duffy blood group, Mauritania, polymorphism, malaria

## Abstract

**Background:**

Duffy blood group polymorphisms are important in areas where *Plasmodium vivax *is present because this surface antigen is thought to act as a key receptor for this parasite. In the present study, Duffy blood group genotyping was performed in febrile uninfected and *P. vivax*-infected patients living in the city of Nouakchott, Mauritania.

**Methods:**

*Plasmodium vivax *was identified by real-time PCR. The Duffy blood group genotypes were determined by standard PCR followed by sequencing of the promoter region and exon 2 of the Duffy gene in 277 febrile individuals. Fisher's exact test was performed in order to assess the significance of variables.

**Results:**

In the Moorish population, a high frequency of the *FYB^ES^/FYB^ES ^*genotype was observed in uninfected individuals (27.8%), whereas no *P. vivax*-infected patient had this genotype. This was followed by a high level of *FYA/FYB*, *FYB/FYB*, *FYB/FYB^ES ^*and *FYA/FYB^ES ^*genotype frequencies, both in the *P. vivax*-infected and uninfected patients. In other ethnic groups (Poular, Soninke, Wolof), only the *FYB^ES^/FYB^ES ^*genotype was found in uninfected patients, whereas the *FYA/FYB^ES ^*genotype was observed in two *P. vivax*-infected patients. In addition, one patient belonging to the Wolof ethnic group presented the *FYB^ES^/FYB^ES ^*genotype and was infected by *P. vivax*.

**Conclusions:**

This study presents the Duffy blood group polymorphisms in Nouakchott City and demonstrates that in Mauritania, *P. vivax *is able to infect Duffy-negative patients. Further studies are necessary to identify the process that enables this Duffy-independent *P. vivax *invasion of human red blood cells.

## Background

Malaria remains one of the most important parasitic infections in the world, with almost 225 million cases of infection and 0.78 million deaths in 2009, mainly in Africa, Asia and South America [1]. It is caused by infection with one or more of five species of *Plasmodium *parasites. *Plasmodium vivax *is the second most common cause of malaria in the world after *Plasmodium falciparum*, with more than 80 million clinical cases annually. Unlike *P. falciparum*, *P. vivax *rarely causes mortality, but it can potentially lead to severe complications and is thereby responsible for considerable morbidity and economic loss in endemic countries [2-8]. Moreover, *P. vivax *has a wider geographical range, potentially exposing more people to risk of infection (2.85 billion across three continents) [9-11], and it is more difficult to control because of the hypnozoïte forms of the parasite [12,13]. The presence of *P. vivax *in Mauritania was first reported in 1948 [14]. More recently, several studies conducted in Nouakchott, the capital of Mauritania, revealed a high proportion of *P. vivax*, followed by *Plasmodium ovale *and *P. falciparum*; autochthonous malaria cases exist but are relatively uncommon [15-17]. In 2009-2010, the prevalence of *P. vivax *among malaria in children in Nouakchott represented 97.1% [18].

One of the main biological differences between *P. vivax *and other human malaria parasites is that only *P. vivax *merozoites use the human Duffy antigen/chemokine receptor (DARC) to invade red blood cells (RBCs) [19-21]. The Duffy antigen was originally identified as a blood group antigen on the surface of RBCs, but it has since been found to be expressed in endothelial cells and neurons [22-24]. It is implicated in multiple chemokine inflammation, inflammatory diseases, and cancer and might play a role in HIV infection [25-27]. The DARC gene (also referred to as FY or Duffy), located on chromosome 1, comprises two exons and produces a protein that has a glycosylated external N-terminal domain, seven transmembrane domains and a short cytosolic C-terminal domain that is not coupled to G-proteins or other known intracellular effectors [28-33].

DARC has two main variant forms, Fya and Fyb antigens, which differ by a single amino acid (Gly42Asp) in the NH_2 _extracellular domain of the polypeptide and are encoded by the alleles *FYA *and *FYB*, respectively, which are differentiated by a single base substitution (G125A) [34-36]. The *FYA/FYB *frequency shows marked geographic disparities; the *FYB *allele is highly predominant in Africa, while the *FYA *allele is dominant in Asia [37]. The Duffy blood group has four major phenotypes: Fy(a^+^b^+^), Fy(a^+^b^-^), Fy(a^-^b^+^) and Fy(a^-^b^-^). Duffy expression is disrupted by a T to C substitution in the gene's promoter region at nucleotide -33, preventing the binding of the h-GATA-1 erythroid transcription factor and resulting in the null expression of the Duffy gene in erythroid cells only [38-40]. This variant is commonly associated with the *FYB *allele (corresponding to the *FYB^ES ^*allele, ES stands for "erythroid silent"), although the same mutation has been detected and associated with the *FYA *allele in individuals living in *P. vivax*-endemic region of Papua New Guinea (*FYA^ES^*) [41]. The *FYB^ES ^*allele is almost fixed in West and Central Africa, and as a consequence, the Fy(a^-^b^-^) (null) phenotype is predominant among populations of West and Central African descent. This phenotype is rare among Caucasian, Amerindian, Indian and Asian populations. The *FYA^ES ^*mutation is rare and so far appears to be present only in the Melanesian and Tunisian population) [41-44]. Other rare variants have been described, most notably the *FYX *allele, which occurs mainly in Caucasians [45,46] and is characterized by a weak expression of Fyb antigen (Fyb^weak^).

Some authors [47-50] have attributed the *FYX *allele to a single polymorphism of the *FYB *allele (C265T→Arg89Cys) (*FYX1*), while others have indicated two (C265T and G298A→Ala100Thr) (*FYX2*) [51-53] or even three polymorphisms (C265T, G298A and G145T→ Ala49Ser) (*FYX3*) [54]. The point mutation G298A alone did not cause a decrease of the Fyb expression [47]. This allele is also named FYB* in the present study. Eight combinations of alleles (*FYA*, *FYB*, FYB*, *FYX *[*FYX1*, *FYX2 *and *FYX3*], *FYA^ES ^*and *FYB^ES^*) result in 32 different genotypes (Additional files 1 and 2).

Malaria therapy, as well as experimental and epidemiological studies, have shown that erythrocyte Duffy blood group negative individuals, mostly of African ancestry, are resistant to *P. vivax *infection [21]. However, several reports have provided evidence for *P. vivax *infections among Duffy-negative patients [55-59], suggesting that there are *P. vivax *strains that have acquired a Duffy-independent mechanism of erythrocyte invasion. Little is known about the frequency of Duffy polymorphisms in Mauritanian populations, especially in *P. vivax*-infected individuals. The objective of the present study was to evaluate the Duffy blood group allelic and genotype frequencies in the city of Nouakchott and to compare these frequencies between *P. vivax-*infected and uninfected febrile patients.

## Methods

### Study populations

This study was conducted in the capital and largest city of Mauritania, Nouakchott, which is located on the Atlantic coast of the Sahara Desert (18°.11'N; 16°.16'W). The city is divided into nine districts and consists of approximately 800,000 inhabitants. Nouakchott features an arid climate with a short wet season extending from July to September. The city has five hospitals and eleven health centres. Between 2007 and 2009, Lekweiry *et al *conducted a preliminary study on the incidence of malaria in Nouakchott [17].

Capillary blood samples from 439 febrile outpatients from all Nouakchott districts who were seen in the two main hospitals of the city (National Hospital and Chiekh Zayed Hospital) and in the District Health Center of Teyarett were collected onto Whatman 3 MM filter paper. A subset of 277 patients were enrolled in this study to evaluate Duffy blood group. Of these 277 patients, 110 had a positive *P. vivax *diagnosis and 167 were not infected with *Plasmodium *but their individual data on the place of residence and/or ethnic group membership were available.

### Consent

This study was reviewed and approved by the Mauritanian National Ethics Committee.

### Genomic DNA extraction

Blood samples were spotted onto Whatman 3 MM filter paper, dried, and stored at room temperature until use. DNA was extracted with the MagMAX™-96 DNA Multi-Sample Kit according to the manufacturer's instructions using a MagMAX™ Express-96 Magnetic Particle Processor (Applied Biosystems, Courtaboeuf, France).

### Identification of *Plasmodium *species by real-time PCR

*Plasmodium *detection was performed by real-time LightCycler^® ^PCR (Roche, Meylan, France). The following oligonucleotides primers and probes designed with Primer Express software v2.0 (Applied Biosystems) were used: forward-5'-TTTATGTATTGGTATAACATTCGG-3', reverse-5'-GGCAAATAACTTTATCATAGAATTGAC-3' and probe-5'-FAM- TACACTACCAACACATGGGGCTACAAGAGGT-BBQ-3' for *P. falciparum *aquaglyceroporin gene (AJ413249); forward-5'-GTGGCCGCCTTTTTGCT-3', reverse-5'-CCTCCCTGAAACAAGTCATCG-3' and probe-5'-HEX- CATCTACGTGGACAACGGGCTCAACA-BHQ1-3' for *P. vivax *enoyl-acyl carrier protein reductase gene (AY423076); forward- 5'-GAGGAATGGTCACCATGTAGTGT-3', reverse-5'-CAAATTTCAGTTTCAAGGTCACTTAA-3' and probe-5'-HEX- ATTTTTTGCATCAACCTTTCTTCTAGCCC -BHQ1-3' for *Plasmodium malariae *circumsporozoïte gene (S69014); and forward-5'-CCAAGCCCAGATAATAAGGAAGGT3', reverse-5'-TTCGTGCACTTCAACTTACATTCAGT-3' and probe-5'-FAM-TTATTGTCCTCTGGGTTTGGAACTTTGCC-BBQ-3' for *P. ovale *P25 ookinete surface protein gene (AB074973) (Eurogentec, Angers, France). Each parasite species was detected separately. Individual PCR amplifications were carried out using 4 μl of 5× concentrate Master Mix (LightCycler^® ^TaqMan^® ^Master, Roche), 0.8 μM of each primer, 0.1 μM of probe and 5 μL of template DNA in a final volume of 20 μL. The thermal cycling conditions were 95°C for 10 min and 45 cycles of 95°C for 10 sec and 60°C for 30 sec, followed by a cooling step of 40°C for 30 sec. For each PCR run, two negative controls (water and human DNA) and a positive control (DNA from each species) were used. Fluorescence acquisition was performed at the end of each extension step.

### Duffy blood group genotyping

Duffy blood group genotypes were assessed using PCR amplification of the human Duffy antigen/chemokine receptor gene (NG_011626.1) followed by sequencing. The promoter region that flanks the GATA box motif (a fragment of 392 bp) was amplified using the following primers, which were designed with the NCBI/Primer-BLAST online tool [60]:

forward-5'-CCCAAGGCCAGTGACCCCCATA-3' and reverse-5'-AGAGGGAGCTAGGAGGCTAGCAT-3' (Eurogentec). To determine the Duffy RBC polymorphism, a 541-bp fragment spanning part of intron and exon 2 was amplified using the following primers (also designed with the NCBI/Primer-Blast online tool [60]): forward-5'-CCTGCAGAGACCTTGTTCTCCCAC-3' and reverse-5'-AGCAGCAAAGCCTGGGCAAAGG-3' (Eurogentec).

The reaction mixture for both PCR amplifications included 10 μl of genomic DNA, 2.5 μl of 10× reaction buffer (Eurogentec), 0.5 μM of each primer, 200 μM of deoxynucleoside triphosphate mixture (dGTP, dATP, dTTP and dCTP) (Euromedex, Souffelweyersheim, France), 1.5 mM of MgCl_2 _and 2.5 units of RedGoldStar^® ^DNA polymerase (Eurogentec) in a final volume of 25 μL. The thermal cycler (T3 Biometra, Archamps, France) was programmed as follows: an initial 94°C incubation for 2 min followed by 40 cycles of 94°C for 30 sec, 58°C for 30 sec and 72°C for 25 sec for the promoter region and 40 cycles of 94°C for 30 sec, 58°C for 30 sec and 72°C for 35 sec for the segment covering part of intron and exon 2. A final 5-min extension step was performed at 72°C for both regions. The PCR products were loaded on 1.5% agarose gel containing 0.5 μg/mL ethidium bromide. Amplicons were purified using the QIAquick 96 PCR BioRobot Kit and an automated protocol on the BioRobot 8000 workstation (Qiagen, Courtaboeuf, France). The purified fragments were sequenced using the BigDye Terminator v3.1 Cycle Sequencing Kit (Applied Biosystems) using the primers described above. The sequencing reaction products were purified using the BigDye XTerminator^® ^Purification Kit (Applied Biosystems) in accordance with the manufacturer's instructions. The purified products were sequenced using an ABI Prism 3100 analyser (Applied Biosystems). Sequences were analysed using Vector NTI advance™ software (version 11, Invitrogen, Cergy Pontoise, France).

### Statistical analysis

Fisher's exact test was used to compare the proportions of Duffy genotypes in relation to *P. vivax *infection and ethnic origin (GraphPad Prism v5.01). The significance level was fixed at *P *< 0.05.

## Results

### *Plasmodium *species diagnosis

The results obtained by real-time PCR were in accordance with the previous data obtained for species diagnosis (Malaria Rapid Diagnostic Test, microscopy and nested PCR performed by [17]). Of 277 outpatients, 110 were positive for *P. vivax*. These patients came from various Nouakchott districts (Additional file 3).

### Duffy genotypes in febrile uninfected patients and *P. vivax*-infected patients from Nouakchott

The promoter region and exon 2 of the Duffy gene from each sample selected for the study were amplified and sequenced (Additional file 3). A comparison of Duffy genotypes, phenotypes and allele frequencies according to the ethnic groups between *P. vivax*-infected and malaria-free patients is presented in Tables 1 and 2. The Moorish population represented the majority of patients (83%). Only a few patients belonged to the other ethnic groups: Poular (4%), Soninke (1%) and Wolof (1%). Information on ethnic origin was not available for some patients (11%), but these patients were included in the study because they were positive for *P. vivax*. The complete sequence of the Duffy gene was obtained for 258 patients (93%).

**Table 1:** Comparison of Duffy genotypes among uninfected and *Plasmodium vivax*-infected patients in ethnic groups from Nouakchott, Mauritania.

ethnic groups	Duffy		number of uninfected patients No = 167 (%[95%CI])	number of *P. vivax*-infected patients No = 110 (%[95%CI])	p-value
	genotypes	predicted phenotypes			
Moor	*FYA/FYA*	positive	11 (7.6 [3.9-13.3])	3 (3.8 [0.8-11.0])	0.388
	*FYA/FYB*	positive	24 (16.6 [11.0-23.8])	22 (28.6 [18.8-40.0])	0.0548
	FYA/FYB*	positive	5 (3.5 [1.1-7.9])	1 (1.3 [0.0-7.0])	0.6674
	*FYA/FYB^ES^*	positive	19 (13.2 [8.1-19.8])	16 (20.8 [12.4-31.5])	0.1757
	FYB*/FYB^ES^	positive	0 (0)	1 (1.3 [0.0-7.0])	0.3484
	*FYB/FYB*	positive	22 (15.3 [9.8-22.2])	16 (20.8 [12.4-31.5])	0.3504
	FYB/FYB*	positive	2 (1.4 [0.2-4.9])	2 (2.6 [0.3-9.1])	0.612
	*FYB/FYB^ES^*	positive	21 (14.6 [9.3-21.4])	16 (20.8 [12.4-31.5])	0.2599
	*FYB^ES^/FYB^ES^*	negative	40 (27.8 [20.6-35.9])	0 (0)	**< 0.0001**^+^

Poular	*FYB^ES^/FYB^ES^*	negative	8 (100)	0 (0)	ND

Soninke	*FYA/FYB^ES^*	positive	0 (0)	2 (100)	0.4667
	*FYB^ES^/FYB^ES^*	negative	2 (100)	0 (0)	0.4667

**Wolof**	***FYB^ES^/FYB^ES^***	**negative**	**1 (100)**	**1 (100)**	**ND**

Unknown	*FYA/FYB*	positive	0 (0)	6 (26.1 [10.2-48.4])	ND
	FYA/FYB*	positive	0 (0)	1 (4.3 [0.1-21.9])	ND
	*FYA/FYB^ES^*	positive	0 (0)	2 (8.7 [1.1-28.0])	ND
	*FYB/FYB*	positive	0 (0)	1 (4.3 [0.1-21.9])	ND
	*FYB/FYB^ES^*	positive	0 (0)	13 (56.6 [34.5-76.8])	ND

ND	ND	ND	12	7	ND

^+ ^Fisher's Exact Test

ND: non determined

**Table 2:** Comparison of allelic frequencies of the Duffy Blood Group System among uninfected and *Plasmodium vivax*-infected patients in ethnic groups from Nouakchott, Mauritania.

ethnic groups	Alleles	Allelic frequencies (%[95%CI])		p-value
		uninfected patients	*P. vivax*-infected patients	
Moor	*FYA*	24.3 [19.5-29.7]	29.2 [22.2-37.1]	0.3058
	*FYB*	31.6 [26.3-37.3]	46.8 [38.7-55.0]	**0.0019^+^**
	FYB*	2.4 [1.0-4.9]	2.6 [0.7-6.5]	1
	*FYB^ES^*	41.7 [35.9-47.6]	21.4 [15.2-28.8]	**< 0,0001^+^**

Poular	*FYB^ES^*	100	0	ND

Soninke	*FYA*	0	100	ND
	*FYB^ES^*	100	0	0.0667

Wolof	*FYB^ES^*	100	100	ND

Unknown	*FYA*	0	19.6 [9.4-33.9]	ND
	*FYB*	0	45.7 [30.9-61.0]	ND
	FYB*	0	21.7 [0.1-11.5]	ND
	*FYB^ES^*	0	32.6 [19.5-48.0]	ND

^+ ^Fisher's Exact Test

ND: non determined

In the Moorish population, the prevalence rate of the *FYB^ES^/FYB^ES ^*genotype (Fy(a^-^b^-^) phenotype) was 27.8% (n = 40) and 0% among uninfected and *P. vivax*-infected individuals, respectively (p < 0.0001, Fisher's exact test). This was followed by a high level of *FYA/FYB*, *FYB/FYB*, *FYB/FYB^ES ^*and *FYA/FYB^ES ^*genotype frequencies (Fy(a^+^b^+^), Fy(a^-^b^+^), Fy(a^-^b^+^) and Fy(a^+^b^-^) phenotypes, respectively) in both *P. vivax*-infected and uninfected patients. Low frequencies were detected for the *FYA/FYA*, FYA/FYB*, FYB*/FYB^ES ^and FYB/FYB* genotypes (Fy(a^+^b^-^), Fy(a^+^b+), Fy(a^-^b^+^) and Fy(a^-^b^+^) phenotypes, respectively) in both infected and uninfected patients. In the other ethnic groups (Poular, Soninke and Wolof), only the *FYB^ES^/FYB^ES ^*genotype was found in uninfected patients, whereas the *FYA/FYB^ES ^*genotype was observed in two *P. vivax*-infected patients, Soninke ethnic.

One *P. vivax*-infected patient presented the *FYB^ES^/FYB^ES ^*genotype, resulting in a Duffy-negative phenotype. This patient was a two-year-old female, belonging to the Wolof ethnic group and living in the district of Dar Naim.

## Discussion

Recent reports on *P. vivax *infections suggest that this parasite may be evolving and adapting to new epidemiological contexts, becoming not only more virulent but also more frequent in countries where the incidence has traditionally been low [9-12,61,62]. The evaluation of Duffy blood group polymorphisms is important in areas where *P. vivax *prevails, as the Duffy antigen serves as a receptor on the surface of RBCs. Until now, few studies have reported the presence of *P. vivax *in Mauritania [15,17,18,29,63] and only one study assessed the distribution of Duffy polymorphisms in Nouakchott [64]. In the present work, the evaluation of Duffy blood group genotypes was undertaken in diverse/multiple human populations that included *P. vivax*-infected, uninfected, Duffy-positive and Duffy-negative people to *i) *assess the Duffy gene polymorphism within a cosmopolitan African community and *ii) *determine whether *P. vivax *is able to penetrate into RBCs in Duffy-negative patients, who have been thought to be resistant to *P. vivax *infection [21].

The Mauritanian population has a highly heterogeneous ethnic composition. It is primarily constituted of Moors (an ethnicity with a mix of Arab and Berber ancestry) who live in the North of the country and various black ethnic groups, including Soninke, Wolof and Poular, in the South. Duffy gene polymorphism among different ethnic groups is a characteristic of this blood system and has been used as a marker of ethnic composition as well as an indicator of the evolution of human populations. In 1986, Lepers *et al *undertook the study of Duffy blood group in 107 individuals belonging to different ethnic groups and residing in Nouakchott [64]. In the overall population, 27% of the individuals were Duffy-positive, whereas the others were Duffy-negative. The proportion of Duffy- positive individuals differed according to the ethnic groups: 54% of Moors were Fy^+^, while only 2% of black ethnic groups were Fy^+^.

In the current study, slight differences were observed in the global population: 78% of the individuals were Fy^+ ^and 22% were Fy^-^. The *FYA/FYB *genotype was the most common, followed by the heterozygotes *FYA/FYB^ES ^*and *FYB/FYB^ES ^*and the homozygous *FYB *alleles. It should be noted that no patient had the phenotype Fy(a^+^b^weak^) or Fy(a^-^b^weak^), as the allele *FYX *was not present in the population.

When compared to other North African populations, the frequencies of *FYA *and *FYB *alleles are similar to that observed in the Tunisian people [44,65], while the allelic frequency of *FYB^ES ^*and the lack of *FYX *are similar to what is observed in Morocco [66]. Overall, *FYA *and *FYB *alleles are mainly represented in Europe, while the allele *FYB^ES ^*is predominant in Africa [37].

The presence of *P. vivax *in Mauritania was first reported in 1948 [14] and confirmed in two recent studies suggesting autochthonous *P. vivax *transmission in some patients who had never travelled outside Nouakchott [15,17]. RBCs of Duffy-negative individuals seem to be naturally resistant to invasion by the *P. vivax *human malaria parasite [21]. The present study describes for the first time that one Duffy-negative patient living in Nouakchott, *i.e*., in North Africa, was infected with *P. vivax*. The identification of *P. vivax *was performed by real-time PCR, and the Duffy genotypes were determined by sequencing, making it unlikely that a parasite other than *P. vivax *was involved.

Our data thereby confirmed the suspicion of some authors, who also believe that *P. vivax *could be evolving to use receptors other than Duffy to invade erythrocytes in patients in Brazil [55,56], Kenya [59], and more recently, in Madagascar [57], Angola and Equatorial Guinea [58]. As suggested in previous studies [57,58], Duffy-positive individuals may serve as reservoirs for *P. vivax*, allowing this parasite to infect hepatocytes of Duffy-negative individuals and select for new *P. vivax *strains with the capacity to invade Duffy-negative erythrocytes.

## Conclusions

Further analyses are needed to understand the dynamics of the Duffy gene and its possible contribution as a modulator in the susceptibility to malaria. The data obtained in the present study emphasize the importance of the evaluation of Duffy blood group genotypes in *P. vivax *malaria endemic areas. The results of the present study support the hypothesis that Duffy-negative individuals from North Africa could be infected by *P. vivax *and that this parasite may be rapidly evolving to use other receptors than Duffy to invade the erythrocytes. Further longitudinal studies on *P. vivax *and host-parasite interactions are required to test the validity of these hypotheses. Furthermore, a better understanding of the alternative pathways used by *P. vivax *to invade human RBCs should become a research priority.

## Competing interests

The authors declare that they have no competing interests.

## Authors' contributions

SB, NW, LKB, JEH, MSOAS, BP, JFT and CR conceived and designed the experiments. KML and NW performed the genotyping of Duffy gene and the diagnosis of *Plasmodium vivax*. HB, AOMSB, SB and KML contributed to reagents/materials/analysis tools. SB, NW and HB analysed the data. NW, SB, LKP and BP wrote the paper. All authors read and approved the final manuscript.

## Supplementary Material

Additional file 1 **Duffy blood group nomenclature**. Duffy alleles and their corresponding genotype and phenotypic and expression. Click here for file

Additional file 2 Phenotype expression relative to 32 different genotypes possible from eight known Duffy alleles (*FYA*, *FYB*, FYB*, *FYX1*, *FYX2*, *FYX3*, *FYA^ES ^*and *FYB^ES^*). Click here for file

Additional file 3 **Individual data, *Plasmodium *diagnosis and Duffy blood group genotypes for the patients selected in the study**. Click here for file
