# Supplementary material for: Vivax malaria in Mauritania includes infection of a Duffy-negative individual
Source: Malar J. 2011 Nov 3;10:336. doi: 10.1186/1475-2875-10-336 (PMC3228859; doi:10.1186/1475-2875-10-336)
Supplement: Additional file 1 — Duffy blood group nomenclature. Duffy alleles and their corresponding genotype and phenotypic and expression. [file 1475-2875-10-336-S1.DOC]

# Additional file 1: Duffy blood group nomenclature

| **Allele** | **nt-33** | **nt125** | **nt145** | **nt265** | **nt298** | **RBC phenotype** | **References** |
| --- | --- | --- | --- | --- | --- | --- | --- |
| *FYA* | T | G | G | C | G | Fya^+^ | [35, 36, 39, 48] |
| *FYB* | T | A | G | C | G | Fyb^+^ | [35, 36, 39, 48] |
| *FYB** | T | A | G | C | A | Fyb^+^ | [47, 54] |
| *FYA^ES^* | C | G | G | C | G | Fya^-^ | [41] |
| *FYB^ES^* | C | A | G | C | G | Fyb^-^ | [40, 48] |
| *FYX1* | T | A | G | T | G | Fyb^weak^ | [47-50] |
| *FYX2* | T | A | G | T | A | Fyb^weak^ | [52-53] |
| *FYX3* | T | A | T | T | A | Fyb^weak^ | [54] |

Duffy alleles and their corresponding genotype and phenotypic expression

nt, nucleotide; RBC, red blood cell; ES, erythrocyte silent, attributed to a T to C mutation at nucleotide −33 in the Duffy gene promoter; * nt298 change alone does not lead to a decreased expression of Fyb; Point mutations in *FYX1* (nt265 alone), *FYX2* (nt265+nt298) and *FYX3* (nt265+nt298+nt145) alleles cause a decrease of Fyb expression.
