# Supplementary material for: Vivax malaria in Mauritania includes infection of a Duffy-negative individual
Source: Malar J. 2011 Nov 3;10:336. doi: 10.1186/1475-2875-10-336 (PMC3228859; doi:10.1186/1475-2875-10-336)
Supplement: Additional file 2 — Phenotype expression relative to 32 different genotypes possible from eight known Duffy alleles (FYA, FYB, FYB*, FYX1, FYX2, FYX3, FYAES and FYBES). [file 1475-2875-10-336-S2.DOC]

**Additional file 2: Phenotype expression relative to 32 different genotypes possible from eight known Duffy alleles (*FYA*, *FYB*, *FYB**, *FYX1*, *FYX2*, *FYX3*, *FYA^ES^* and *FYB^ES^***)

| **RBC phenotype** | **genotype** | **antigen** | **nt-33** | **nt125** | **nt145** | **nt265** | **nt298** |
| --- | --- | --- | --- | --- | --- | --- | --- |
| (a^+^b^+^) | *FYA/FYB* | Fya^+^Fyb^+^ | T/T | G/A | G/G | C/C | G/G |
|  | *FYA/FYB** |  | T/T | G/A | G/G | C/C | G/A |
| (a^+^b^-^) | *FYA/FYA* | Fya^+^ | T/T | G/G | G/G | C/C | G/G |
|  | *FYA/FYB^ES^* |  | T/C | G/A | G/G | C/C | G/G |
|  | *FYA/FYA^ES^* |  | T/C | G/G | G/G | C/C | G/G |
| (a^+^b^weak^) | *FYA/FYX1* | Fya^+^Fyb^weak^ | T/T | G/A | G/G | C/T | G/G |
|  | *FYA/FYX2* |  | T/T | G/A | G/G | C/T | G/A |
|  | *FYA/FYX3* |  | T/T | G/A | G/T | C/T | G/A |
| (a^-^b^+^) | *FYB/FYB* | Fyb^+^ | T/T | A/A | G/G | C/C | G/G |
|  | *FYB/FYB** |  | T/T | A/A | G/G | C/C | G/A |
|  | *FYA^ES^/FYB* |  | C/T | G/A | G/G | C/C | G/G |
|  | *FYA^ES^/FYB** |  | C/T | G/A | G/G | C/C | G/A |
|  | *FYB/FYB^ES^* |  | T/C | A/A | G/G | C/C | G/G |
|  | *FYB*/FYB^ES^* |  | T/C | A/A | G/G | C/C | A/G |
|  | *FYB/FYX1* |  | T/T | A/A | G/G | C/T | G/G |
|  | *FYB/FYX2* |  | T/T | A/A | G/G | C/T | G/A |
|  | *FYB/FYX3* |  | T/T | A/A | G/T | C/T | G/A |
|  | *FYB*/FYX1* |  | T/T | A/A | G/G | C/T | A/G |
|  | *FYB*/FYX2* |  | T/T | A/A | G/G | C/T | A/A |
|  | *FYB*/FYX3* |  | T/T | A/A | G/T | C/T | A/A |
| (a^-^b^weak^) | *FYA^ES^/FYX1* | Fyb^weak^ | C/T | G/A | G/G | C/T | G/G |
|  | *FYA^ES^/FYX2* |  | C/T | G/A | G/G | C/T | G/A |
|  | *FYA^ES^/FYX3* |  | C/T | G/A | G/T | C/T | G/A |
|  | *FYB^ES^/FYX1* |  | C/T | A/A | G/G | C/T | G/G |
|  | *FYB^ES^/FYX2* |  | C/T | A/A | G/G | C/T | G/A |
|  | *FYB^ES^/FYX3* |  | C/T | A/A | G/T | C/T | G/A |
|  | *FYX1/FYX2* |  | T/T | A/A | G/G | T/T | G/A |
|  | *FYX1/FYX3* |  | T/T | A/A | G/T | T/T | G/A |
|  | *FYX2/FYX3* |  | T/T | A/A | G/T | T/T | A/A |
| (a^-^b^-^) | *FYA^ES^/FYA^ES^* | no antigen | C/C | G/G | G/G | C/C | G/G |
|  | *FYB^ES^/FYB^ES^* |  | C/C | A/A | G/G | C/C | G/G |
|  | *FYA^ES^/FYB^ES^* |  | C/C | G/A | G/G | C/C | G/G |

nt, nucleotide; RBC, red blood cell; ES, erythrocyte silent.
